# The Bad Case of the Baker

**Published:** 1913-03-22

**Authors:** 


### The Hospital

The Workers' Newspaper of Administrative Medicine and Institutional Life, Administration, National Insurance and Health.

No. 1394, Vol. LIII. SATURDAY, MARCH 22, 1913. PRICE ONE PENNY.

### THE BAD CASE OF THE BAKER.

The recent trouble in the baking trade in London has called the attention of the public to the grave dangers, both to the community and to individuals, that are still unfortunately the concomitants of the baker who works in insanitary surroundings. It is unhappily the fact that while the public, in the agitation of a possible strike, sympathises or deplores, it speedily forgets, when the reaction has set in, the lessons that were forced upon it. Again, there is, not unnaturally, a tendency to make light of strikers' alleged grievances, and to put every movement of the nature of a strike down to the interference of interested agitators. But in this case an impartial survey of the whole position of the baking trade can scarcely warrant the assumption that the baker has nothing to complain of or that the public has no interest in seeing that his complaint is redressed. The hours of work that are his are long and are spent very often in an environment that saps his vitality to the utmost. He works, at the best, in an atmosphere which is suggestive of a stokehold, with the same accompaniment of dust. It is true an up-to-date modern bakehouse, of the type with which large hospitals have equipped themselves, is an immense advance upon the old insanitary underground bakehouse which still stands in many parts of the country. But even under these almost ideal conditions the occupation of baking is not a conspicuously healthy one. The mortality among bakers is very high, and the causes of death, it is instructive to note, are essentially causes which are directly or indirectly due to occupation. Pulmonary disease, in the form of phthisis or pneumonia, head the list of bakers' diseases, while rheumatism and chronic arthritis are perhaps equally common.

It is, probably, no longer true that baking is responsible for certain deformities, since much of the weight lifting and carrying is done by machinery. Yet the old term "baker-legged" remains with us as a synonym for "bandy-legged," and although it is almost obsolete it should serve to draw attention to the fact that the baker's life is not a healthy one, even under the best conditions. And that many carry on their trade in premises which are the reverse of ideal there can be no question whatever. The revelations—revelations to the uninitiated public that is, for the Medical Officers of Health know perfectly well what conditions obtain in certain establishments, though they are, in the present state of the law, unable to alter them—have made it clear that there are many bakehouses which, by their insanitary state and their lack of conveniences for the men, enhance the danger to which the workers are exposed. One of the main wants is undoubtedly adequate cleansing and bathing accommodation. In an occupation which entails so much dust and perspiration as does that of the baker it is essential that every means shall be given to the workman to keep himself as clean and as wholesome as is humanly possible.

So far we have alluded chiefly to the dangers which the baking trade possesses for the individual workman. There yet remains the equally important aspect of the dangers to the community. Where an article of daily diet so generally used as bread is prepared by men who are potential factors of infection, owing to disease or want of cleanliness, it is imperative that every precaution should be exercised in safeguarding the public. Something has been said before now with regard to the insanitary methods of bread distribution; we have all heard of the dirty glove of the baker's boy, and there is even, we believe, a record of bacteriological investigation of the pathogenic flora which that relic of pre-aseptic times furnishes. But far too little attention has been paid to the conditions under which the working baker carries on his trade, to his bakery, his work-room, and his habits while at work. These are matters with which the public might worthily concern itself, in its own interest as well as in the interests of the trade. Possibly further legislative safeguards are desirable, but the law already gives a certain measure of supervisory power to local bodies to make their own by-laws with regard to some important points. Now that there is a further possibility that the Local Government Board will take up the matter and issue regulations, it behoves the public to keep a watchful eye on the conditions of the trade, and to insist that the bread which is supplied to it shall be baked under sanitary conditions in sanitary houses which afford every convenience to the workmen to keep themselves clean and healthy.

